# Supplementary material for: Ultraviolet supercontinuum generation driven by ionic coherence in a strong laser field
Source: Nat Commun. 2022 Jul 14;13:4080. doi: 10.1038/s41467-022-31824-0 (PMC9283425; doi:10.1038/s41467-022-31824-0)
Supplement: Supplementary file 1 — Supplementary Information [file 41467_2022_31824_MOESM1_ESM.pdf]

Supplementary Information:

Ultraviolet supercontinuum generation driven by ionic coherence in a strong laser field

Hongbin Lei[1], Jinping Yao[2,*], Jing Zhao[1], Hongqiang Xie[2,3], Fangbo Zhang[2], He Zhang[2], Ning Zhang[2], Guihua Li[4], Qian Zhang[1], Xiaowei Wang[1], Yan Yang[1], Luqi Yuan[5], Ya Cheng[2], and Zengxiu Zhao[1,*]

[1]Department of Physics, National University of Defense Technology, Changsha 410073, China.

[2]State Key Laboratory of High Field Laser Physics, Shanghai Institute of Optics and Fine Mechanics, Chinese Academy of Sciences, Shanghai 201800, China.

[3]School of Science, East China University of Technology, Nanchang 330013, China.

[4]School of Science, East China Jiaotong University, Nanchang 330013, China.

[5]State Key Laboratory of Advanced Optical Communication Systems and Networks, School of Physics and Astronomy, Shanghai Jiao Tong University, Shanghai 200240, China.

[*]Corresponding authors: jinpingmrg@163.com; zhao.zengxiu@gmail.com

Supplementary Note 1: Basic parameters of theoretical simulation

In our simulation, the Hamiltonian $H(t)$ is given by

$$\begin{bmatrix} \omega_B^{v_1'} & \cdots & 0 & -d(Bv_1',Xv_1)E(t) & \cdots & -d(Bv_1',Xv_m)E(t) \\ \vdots & \ddots & \vdots & \vdots & \ddots & \vdots \\ 0 & \cdots & \omega_B^{v_m'} & -d(Bv_m',Xv_1)E(t) & \cdots & -d(Bv_m',Xv_m)E(t) \\ -d(Bv_1',Xv_1)E(t) & \cdots & -d(Bv_m',Xv_1)E(t) & \omega_X^{v_1} & \cdots & 0 \\ \vdots & \ddots & \vdots & \vdots & \ddots & \vdots \\ -d(Bv_1',Xv_m)E(t) & \cdots & -d(Bv_m',Xv_m)E(t) & 0 & \cdots & \omega_X^{v_m} \end{bmatrix} \tag{S1}$$

where $E(t) = E_0(t)\cos(\omega_l t)$ is the electric field of the driver laser, $E_0(t)$ is the Gaussian envelope, ω_n^v is the field-free eigenenergy of the electronic-vibrational state, $d(Bv',Xv) = \int \Phi_{Bv'}(R) d_{BX}(R) \Phi_{Xv}^*(R)\, dR$ is the transition dipole moment (TDM), and the vibrational wave functions are given by $\Phi_{Bv'}(R)/R$ and $\Phi_{Xv}(R)/R$. The values of TDMs and the radiation wavelengths for various $B(v') - X(v)$ transitions [1,2] are given in Supplementary Table 1. The transient transition energies of various electronic-vibrational transitions are obtained by calculating the cycle-averaged transient eigenvalues of Hamiltonian $H(t)$.

$B(v') - X(v)$	Wavelength (nm)	TDM (a. u.)
$0-0$	391	0.597
$1-0$	358	0.373
$2-0$	331	0.130
$0-1$	428	−0.385
$1-1$	388	0.354
$2-1$	356	0.433
$0-2$	471	0.202
$0-3$	522	0.023

Supplementary Table 1. Radiation wavelengths and TDMs for various $B(v') - X(v)$ transitions of N_2^+. The transition wavelengths[1] are given in units of nm and the dipole moments[2] are given in atomic units.

In the ionization-coupling model (see Eq. (1) in Methods of main text) [3], $\Gamma_{nv} = w(E,\theta)F_{nv}$ is the diagonal matrix of ionization rate from the ground vibrational level of N_2 to the vibrational level v of the electronic state n of N_2^+. Here, $w(E,\theta)$ is the static ionization rate from different orbitals of N_2, and $F_{nv} = \left|\int \Phi_X^{N_2}(R) \cdot \Phi_{nv}^{N_2^+ *}(R)dR\right|^2$ is the Franck-Condon factor corresponding to the transition from the vibrational ground state of N_2 to various vibrational levels of the B or X states of N_2^+, which is given in Supplementary Table 2.

B		X	
State	Franck-Condon	State	Franck-Condon
$v' = 0$	0.891	$v = 0$	0.911
$v' = 1$	0.107	$v = 1$	0.083
$v' = 2$	1.68×10^{-3}	$v = 2$	5.16×10^{-3}
$v' = 3$	2.78×10^{-5}	$v = 3$	2.97×10^{-5}
$v' = 4$	4.11×10^{-6}	$v = 4$	1.74×10^{-6}

Supplementary Table 2. Franck-Condon factors corresponding to the transitions from the vibrational ground state of N_2 to different vibrational levels of the B or X states of N_2^+.

Supplementary Note 2: The induced dipole moment and population dynamics

Supplementary Fig. 1a shows the induced dipole moment $D(t)$ driven by the 1580 nm, 60 fs, 2.0×10^{14} W cm^{-2} laser pulse. Unlike the electric field, the induced dipole moment exhibits an unsymmetrical temporal profile. Owing to the nonlinear dependence of ionization on the laser field strength, the coherent couplings among various ionic states mainly occur in the falling edge of the driver pulse. Besides, the striking oscillations appear after the end of the driver pulse, owing to the free-induction decay (FID) of the $0-0$ and $1-0$ coherences. The quantum beat of the two transitions results in the slow oscillation with the period of ~14 fs. In Supplementary Fig. 1b, we show the time evolution of population variation $P_{\mathrm{IC}} - P_{\mathrm{I}}$, where

P_{IC} and P_{I} represent the population on the corresponding electronic-vibrational state with and without the coupling process, respectively. It can be clearly seen that the coherent coupling results in the decrease of population on the $X(\nu = 0)$ state and the increase of population on other electronic-vibrational levels. This manifests that the population can be transferred among various electronic-vibrational levels via five-photon resonance assisted by Stark shift, as shown in the insets of Fig. 3d-f of main text.

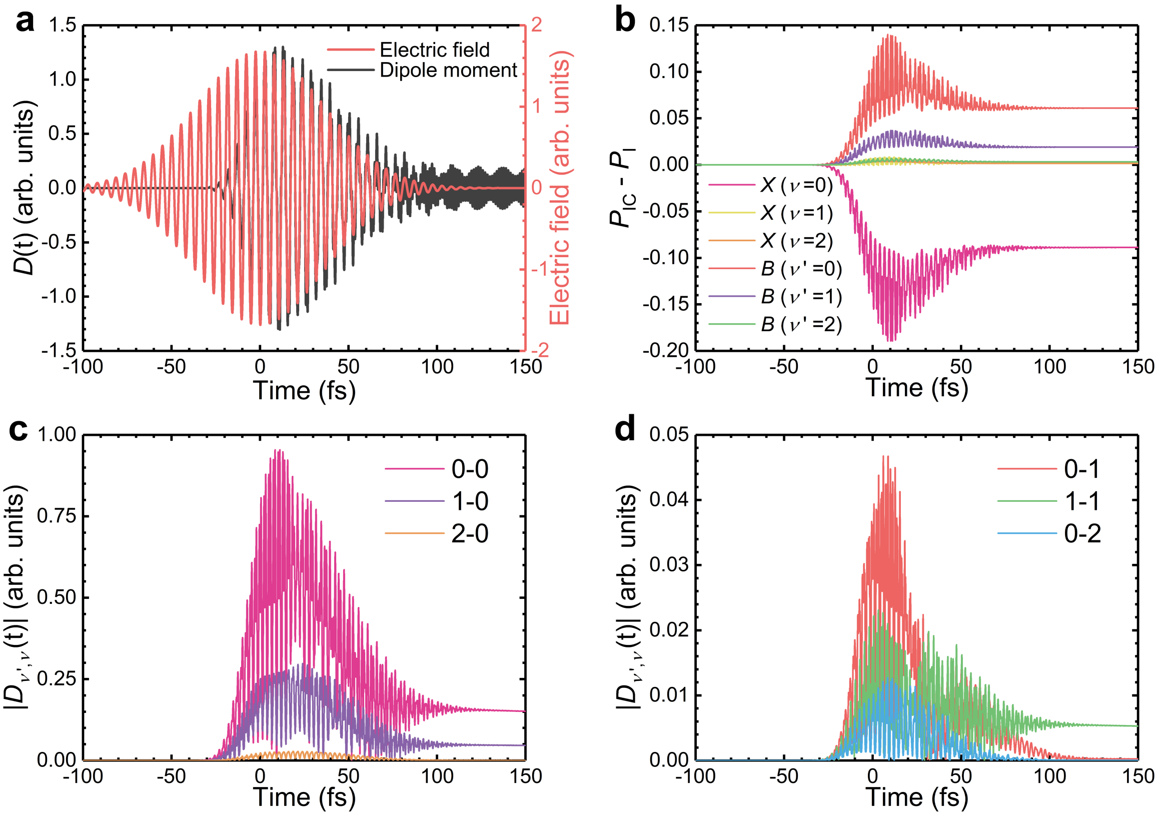

Supplementary Fig. 1. Dynamic evolution of the induced dipole moment and population. **a** The dipole moment induced by the 1580 nm, 60 fs, 2.0×10^{14} W cm^{-2} laser pulse (black line) and the corresponding driver laser field (red line). **b** The population dynamics of the main electronic-vibrational levels. **c**, **d** show amplitudes of six individual dipole moments.

In Supplementary Fig. 1c and d, we show the amplitudes of individual dipole moments $|D_{\nu',\nu}(t)|$ for $0-0$, $1-0$, $2-0$, $0-1$, $1-1$, and $0-2$ transitions. As we can see, the induced dipole moments of $0-0$, $1-0$, and $0-1$ transitions are relatively larger within the driver pulse, and thus they can be considered as the main pathways to induce the laser-assisted continuum emission (LACE). It means that the strong couplings between these

energy levels driven by the intense femtosecond laser pulse. After the end of the drive laser, the $0-0$, $1-0$, and $1-1$ coherences are still remained due to the efficient five-photon resonance couplings, giving rise to the FID signals at the corresponding transition wavelengths. It is noteworthy that since the $X(\nu=1)$ level cannot be efficiently populated via the five-photon resonance coupling, the $0-1$ coherence almost disappears after the laser pulse. However, this level still plays a vital role in promoting Stark shift of the $0-0$ transition and further creating the coherence between two states, as discussed in the main text.

Supplementary References:

[1] Yao, J. et al. Population redistribution among multiple electronic states of molecular nitrogen ions in strong laser fields. *Phys. Rev. Lett.* **116**, 143007 (2016).

[2] Langhoff, S. R. & Bauschlicher, C. W. Theoretical study of the first and second negative systems of N_2^+. *J. Chem. Phys.* **88**, 329-336 (1988).

[3] Zhang, Q. et al. Sub-cycle coherent control of ionic dynamics via transient ionization injection. *Commun. Phys.* **3**, 50 (2020).
