# Supplementary material for: Ultraviolet supercontinuum generation driven by ionic coherence in a strong laser field
Source: Nat Commun. 2022 Jul 14;13:4080. doi: 10.1038/s41467-022-31824-0 (PMC9283425; doi:10.1038/s41467-022-31824-0)
Supplement: Supplementary file 3 — Description of Additional Supplementary Files [file 41467_2022_31824_MOESM3_ESM.pdf]

Description of Additional Supplementary Files

File name: Supplementary Movie 1

Description: The detailed mechanism and dynamic processes of ultraviolet supercontinuum generation in molecular nitrogen ions.
